# Comprehensive analysis of regulatory B Cell related genes in prognosis and therapeutic response in lung adenocarcinoma

**DOI:** 10.3389/fimmu.2025.1595408

**Published:** 2025-07-30

**Authors:** Liangyu Zhang, Jianshen Zeng, Xun Zhang, Menglong Zhang, Yilin Lin, Fancai Lai

**Affiliations:** ^1^ Department of Thoracic Surgery, the First Affiliated Hospital, Fujian Medical University, Fuzhou, China; ^2^ Department of Thoracic Surgery, National Regional Medical Center, the First Affiliated Hospital, Fujian Medical University, Fuzhou, China

**Keywords:** Breg, LUAD, artificial intelligence, prognostic model, immune

## Abstract

**Background:**

Regulatory B cells (Bregs) are a distinct subset of B cells that play a crucial role in regulating immune responses and maintaining immune tolerance in cancerous environments. However, their function in lung adenocarcinoma (LUAD) remains largely underexplored. This study seeks to investigate the roles of Breg-associated genes in the context of LUAD.

**Methods:**

ConsensusClusterPlus package was used to characterize LUAD patients into two clusters. Differentially expressed genes between the two clusters were then used to construct the BREGI using 32 algorithms, including traditional regression, machine learning, deep learning, and 274 different combinations. The training set, TCGA-LUAD, along with SNV and CNV data, was obtained from the TCGA database. Seven external validation sets and one single-cell RNA sequencing set were downloaded from GEO. Data from the TIDE, TCIA, and TIGER websites were curated to assess the effectiveness of immunotherapy.

**Results:**

LUAD patients were divided into two clusters based on 27 Breg-related genes. Patients in Cluster C1 exhibited better prognosis, along with higher immune cell infiltration and immune molecule expression levels, displaying characteristics of a “hot immune” phenotype. The BREGI demonstrated robust predictive power for LUAD patient prognosis across various cohorts. Patients with high BREGI were associated with poor prognosis, higher gene mutation frequencies, a “cold immune” phenotype, and potential resistance to immunotherapy.

**Conclusions:**

Breg-related genes significantly characterize LUAD patients into distinct clusters, and the BREGI demonstrated strong prognostic value, providing new insights for future research on Bregs.

## Introduction

Lung cancer remains a major global health challenge and is among the leading causes of cancer-related morbidity and mortality (1). Non-small cell lung cancer (NSCLC) represents the most common pathological type of lung cancer, accounting for approximately 85% of all cases. Among its subtypes, lung adenocarcinoma (LUAD) is the most prevalent, making it a primary focus of research and clinical intervention (2, 3). Despite significant advancements in therapeutic strategies for LUAD, the prognosis remains poor, with the 5-year overall survival rate still falling below 20% (4). Its high prevalence and devastating impact make it a significant public health threat, underscoring the urgent need for improved prevention, early detection, and treatment strategies.

The tumor microenvironment (TME) in NSCLC is a highly intricate ecosystem composed of immune cells, stromal components, and signaling molecules, all of which critically shape cancer progression and modulate therapeutic responses (5, 6). Within the diverse immune landscape of the TME, B cells have gained recognition as key regulators, orchestrating immune responses that significantly impact tumor development and progression (7, 8). Tumor-infiltrating B cells encompass a diverse spectrum of phenotypes, including naïve, activated, and memory B cells, as well as germinal center B cells and plasma cells (9), each contributing uniquely to the tumor microenvironment. Regulatory B cells (Bregs) constitute a distinct subset of B cells characterized by their potent immunosuppressive functions, playing a vital role in preserving immune tolerance and modulating inflammatory responses (10, 11). Initially identified in autoimmune diseases, Bregs have since been recognized for their pivotal roles in immune regulation across various contexts, including infections, allergic reactions, and transplant tolerance (12). In tumor biology, Bregs have been shown to suppress anti-tumor immune responses, thereby facilitating tumor progression. Moreover, their infiltration into the tumor microenvironment has been correlated with poor prognosis across multiple cancer types, including NSCLC (13–15). Bregs also actively engage with various immune cell populations, including dendritic cells, macrophages, NK cells, and regulatory T cells (Tregs), orchestrating a profoundly immunosuppressive microenvironment that supports tumor immune evasion (16, 17). Previous studies have suggested that within the TME of NSCLC, VISTAχ Bregs may contribute to the suppression of anti-tumor T cell responses through multiple mechanisms. These include the secretion of immunosuppressive cytokines such as IL-10 and TGF-β, as well as direct cell-to-cell interactions mediated by the VISTA-PSGL-1 axis (18). However, a comprehensive evaluation of the prognostic significance of Breg-related genes in NSCLC, particularly in LUAD, remains unexplored.

In our study, we initially curated Breg-related genes from the research conducted by Zhou et al., where Breg-associated genes were identified through the compilation of immunosuppressive gene sets, Breg cell marker genes, and genes differentially expressed between IL10⁻ and IL10χ B cells (19). Based on these Breg-related genes, we performed molecular subtyping of LUAD patients, identifying two distinct clusters that exhibited markedly different TME landscapes and distinct “hot” and “cold” immune phenotypes. Additionally, leveraging the differentially expressed genes between these two clusters, we employed an AI-driven approach to construct a Regulatory B cell related Index (BREGI), which demonstrated superior predictive performance in forecasting LUAD patient prognosis, TME features, and therapeutic efficacy. This study represents the first comprehensive analysis of the roles of Breg-related genes in LUAD, providing critical insights into the functional significance of Bregs and establishing a foundation for future mechanistic studies targeting Bregs in LUAD.

## Materials and methods

### Bulk-RNA sequencing data acquired

The TCGA database (20) provided bulk tumor transcriptomic data for 497 LUAD patients, along with their clinical information, transcriptomic profiles, CNV (Copy Number Variation) and SNV (Single Nucleotide Variants) data. CNV data was analyzed using GISTIC 2.0 (21), while SNV data was processed with the maftools R package (22). External validation was performed using datasets GSE72094, GSE31210, GSE68465, GSE41271, GSE42127, GSE13213 and GSE8894 obtained from GEO (23). The selection of these GEO datasets was based on the following criteria: 1) the inclusion of comprehensive prognostic data for LUAD patients; 2) a sufficiently large sample size; 3) data derived from well-characterized cohorts with consistent inclusion criteria; 4) the availability of detailed molecular and clinical data relevant to our analysis; 5) rigorous quality control measures applied to the dataset and 6) patients didn’t received chemotherapy of other treatments before. Detailed information for these datasets can be found in the supplementary tables (**Supplementary Table S1**). For genes with multiple corresponding probes, the average expression value of these probes was used to represent the gene expression level. The 27 Regulatory B cell (Breg) associated genes were obtained from the study by Zhou et al. (19).

### Processing single-cell RNA-sequencing data

The single-cell RNA-sequencing dataset GSE131907 was acquired from the GEO database. We selected 11 primary tumor samples and 10 brain metastasis samples for analyse. Batch effects were mitigated using the Harmony R package (24). Using the Harmony and Seurat (25) packages, we meticulously performed all necessary procedures, including NormalizeData, FindVariableFeatures, ScaleData, RunPCA, FindNeighbors,RunHarmony, FindClusters, and RunUMAP, ensuring the accuracy of each step. This comprehensive analysis resulted in the acquisition of 52,832 single cells. The AUCell package was utilized to assess gene set activity in single-cell RNA-seq data, identifying cells with active gene sets by calculating the area under the curve (AUC) (26).

### Consensus clustering identified Breg-related clusters

After employing the robust ConsensusClusterPlus package (27) for clustering analysis, we successfully delineated two distinct subgroups among LUAD patients, characterized by the genetic profiles of 27 Breg-related genes. These subgroups exhibited notable disparities in TME compositions.

### Development of a robust regulatory B cell-related gene signature for prognostic prediction in LUAD patients

Differential expression analysis between patients at different Breg-related clusters was conducted using the Limma package, defining differentially expressed genes (DEGs) based on criteria of adjusted P-value (Padj) < 0.001 and absolute log2(Fold-change) > 0.5. To identify key genes among these DEGs, we established the following criteria:

1. Genes must be present in all eight selected datasets to ensure the model’s applicability across different cohorts.
2. Genes exhibit prognostic significance in TCGA, GSE72094, and GSE68465 (P-value < 0.05 in univariate Cox regression analysis), as these three datasets have the largest sample sizes and offer the highest statistical robustness.

To establish a reliable and accurate gene signature associated with regulatory B cells for predicting the prognosis of LUAD patients, we developed an advanced artificial intelligence (AI) framework integrating 32 algorithms from traditional regression, machine learning, and deep learning. By systematically exploring 274 algorithmic combinations, we aimed to achieve both predictive accuracy and model stability.

Our methodological approach encompassed traditional regression models, including stepwise Cox regression, LASSO, Ridge, Elastic Net (Enet), and the Cox Proportional Hazards Model (Coxph). We incorporated ensemble learning techniques such as Random Survival Forest (RSF), Conditional Random Forest (CForest), Oblique Random Survival Forest (obliqueRSF), and Boruta, alongside tree-based algorithms like Recursive Partitioning and Regression Trees (Rpart), Conditional Inference Trees (Ctree), and Ranger.

For boosting-based methods, we implemented Gradient Boosting Machine (GBM), LightGBM, Extreme Gradient Boosting (XGBoost), Gradient Boosting with Component-Wise Linear Models (GLMBoost), Gradient Boosting with Regression Trees (BlackBoost), Generalized Additive Model Boosting (GamBoost), and CoxBoost. Additionally, we integrated Partial Least Squares Regression for Cox (plsRcox), Prediction Analysis for Microarrays (Pamr), and Linear Discriminant Analysis (LDA) for feature selection and dimensionality reduction.

Deep learning-based survival models included DeepHit, DeepSurv, Cox-Time Survival Neural Network (CoxTime), Logistic-Hazard Survival Neural Network (Logistic-Hazard), and PC-Hazard Survival Neural Network (PC-Hazard). We further incorporated Survival Support Vector Machine (Survival-SVM) and parametric survival models such as Survreg and Akritas Conditional Non-Parametric Survival Estimator (Akritas). To enhance variable selection and stability, we employed the Variable Selection-Oriented LASSO Bagging Algorithm (VSOLassoBag).

This comprehensive AI-driven approach provides a robust foundation for constructing an effective gene signature for LUAD prognosis, integrating a diverse range of statistical, machine learning, and deep learning methodologies.

### Functional enrichment analysis

To uncover the biological pathways linked to specific genes, we performed comprehensive enrichment analyses using the R package ClusterProfiler (28). This included Gene Ontology (GO) annotation, Kyoto Encyclopedia of Genes and Genomes (KEGG) pathway analysis, and Gene Set Enrichment Analysis (GSEA).

### Analysis the relationship between BREGI and the TME

The “GSVA” R package (29) was employed to perform single-sample gene set enrichment analysis (ssGSEA), enabling the quantification of enrichment scores for individual gene sets across different samples. In addition, we utilized the ‘IOBR’ package (30) to assess immune cell infiltration levels in LUAD patients by integrating six distinct computational algorithms: quanTIseq, TIMER, EPIC, MCP-counter, ESTIMATE, and xCell. Information on the activation stages of the seven-phase Cancer Immunity Cycle was obtained from the Tracking Tumor Immunophenotype (TIP) database (31). Furthermore, we explored the association between immune-related molecule expression and BREGI.

### Analysis the relationship between BREGI and immunotherapy

The TIDE (Tumor Immune Dysfunction and Exclusion) score, a computational predictor of immune checkpoint blockade (ICB) therapy response in LUAD patients, was obtained from the TIDE website (32), with lower scores indicating higher sensitivity to ICB treatment. Additionally, the Immunophenoscore (IPS) for LUAD patients were retrieved from the TCIA database (33), where a higher IPS score reflects greater responsiveness to ICB therapy. Five immunotherapy cohorts—GSE91061, phs000452, PRJEB23709, phs000452, and PRJNA482620—were obtained from the TIGER database (34). Additionally, another immunotherapy cohort for NSCLC patients (PMID: 37024582) was sourced from the study by Ravi et al. (35). We calculated the BREGI across these cohorts and analyzed the prognostic differences between patients stratified into different BREGI groups.

### Identification of potential drugs targeting high-BREGI patients

To evaluate the therapeutic response of high-BREGI LUAD patients, we integrated data from the GDSC1 database (36) with analyses conducted using the ‘oncoPredict’ package (37). Drug sensitivity was assessed based on IC50 values, where a lower IC50 indicates greater responsiveness to the treatment.

### Cell culture, transfection, and qRT-PCR

The human lung adenocarcinoma cell line A549 was procured from the Cell Bank of the Chinese Academy of Sciences. Cells were maintained at 37°C in a humidified atmosphere with 5% CO2, using DMEM medium (Bioscience, China) supplemented with 10% fetal bovine serum (FBS; Gibco, USA). Small hairpin RNAs targeting TBRG4 (shTBRG4) and non-targeting control shRNA were acquired from Hanheng Biology (Shanghai, China). Transfection of HBLV vector-encoded shTBRG4 and control shRNA was conducted over 24 hours using polybrene reagent, following standard operational procedures. The nucleotide sequences were as follows: negative control (NC): TTCTCCGAACGTGTCACGTAA; shTBRG4: TCAAGCAGCAATGGTACTTAT.​Post-transfection, total RNA was extracted from A549 cells using an RNA isolation kit (Vazyme, China) according to the manufacturer’s protocols. Reverse transcription and PCR amplification were performed with kits obtained from TransGen Biotech (China), adhering to the supplier’s instructions. GAPDH served as the internal reference gene, and relative gene expression was calculated using the 2⁻ΔΔCT method. Primer sequences utilized were: TBRG4 forward 5′-TTCAACAGCCGAAGCAAGGA-3′ and reverse 5′-GGGAGTAGATGCTCGTTCCTTC-3′; GAPDH forward 5′-GGTGTGAACCATGAGAAGTATGA-3′ and reverse 5′-GAGTCCTTCCACGATACCAAAG-3′.

### CCK-8 assay

Cells were plated in 96-well plates at a density of 3,000 cells per well, with 200 μl DMEM medium per well. Culture medium was replenished daily. At each time point, 10 μl CCK-8 solution was added to each well, followed by a 2-hour incubation. Absorbance at 450 nm was subsequently determined using a microplate reader.

### Wound healing assays

Transfected A549 cells were seeded in 6-well plates at 1×10⁵ cells per well. After 24 hours of culture, when cell confluence reached approximately 100%, linear wounds were created in the monolayer using a 10-μl pipette tip. Detached cells were gently removed by PBS washing, and reference marks were made on the plate bottom. Wound areas were imaged at 0 and 24 hours, with quantitative analysis of wound closure performed using ImageJ.

### Transwell migration and invasion assays​

Migration and invasion assays were executed using Transwell chambers (Corning Inc., USA). For invasion assays, chamber inserts were pre-treated with 10 μg Matrigel. A total of 10,000 cells suspended in 200 μl FBS-free DMEM were added to the upper chamber, while 600 μl DMEM containing 10% FBS was placed in the lower chamber. Following 24 hours of incubation at 37°C, cells adhering to the membrane were fixed in paraformaldehyde and stained with hematoxylin. Migrated or invaded cells in the lower chamber were visualized and photographed under a high-power microscope.

### Statistical analysis

Statistical analyses were conducted using R (version 4.1.3). The appropriate test, either the Wilcoxon test or t-test, was used to compare two groups, based on data distribution. Spearman correlation was employed for correlation analyses. Kaplan-Meier survival analysis was performed to assess overall survival differences between groups, and the log-rank test was used to determine statistical significance. To evaluate the prognostic value of BREGI alongside clinicopathological factors, both univariate and multivariate Cox regression analyses were carried out. The CompareC package was used to compare the C-index of BREGI with various models and clinical factors. Statistical significance was defined as *p < 0.05, **p < 0.01, and ***p < 0.001, with “Ns” indicating a p-value ≥ 0.05.

## Results

### Exploring 27 Breg related genes’ significance in LUAD

**Figure 1:**
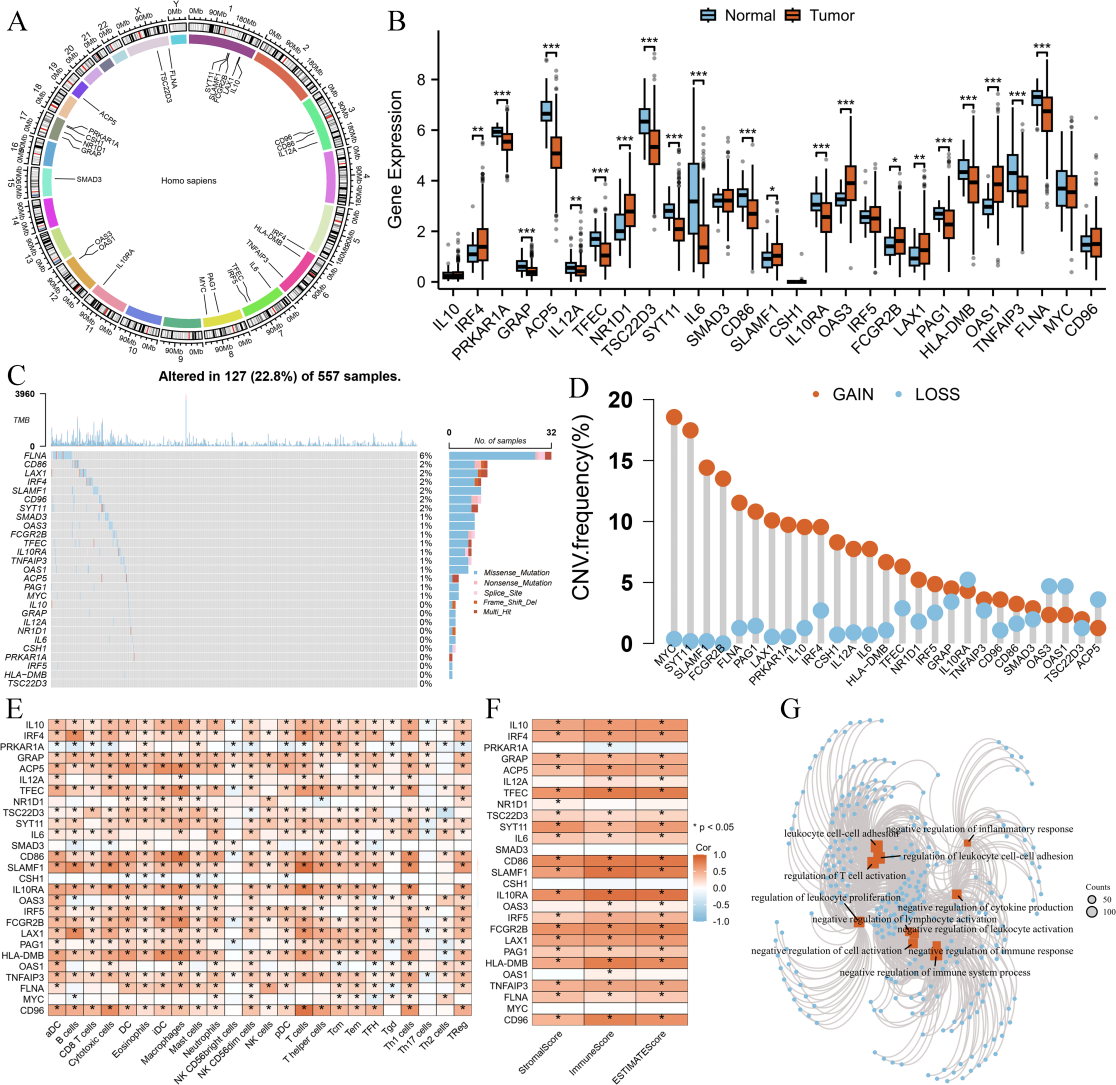
Genomic and immune characteristics of 27 Breg-related genes in LUAD. **(A)** Chromosomal locations of the 27 Breg-related genes. **(B)** Differential expression analysis of the 27 Breg-related genes between normal lung tissues and LUAD tissues **(C, D)** Somatic mutation and Copy number variation **(D)** landscape of the 27 Breg-related genes in LUAD patients. **(E)** Correlation between Breg-related genes and immune cell infiltration levels, as assessed by the single-sample Gene Set Enrichment Analysis (ssGSEA) algorithm in the GSVA package. **(F)** Correlation between Breg-related genes and immune-related scores, calculated using the ESTIMATE algorithm in the IOBR package. **(G)** GO enrichment analysis of the 27 Breg-related genes. ‘*’ means P value is less than 0.05, ‘**’ means P value is less than 0.01, ‘***’ means P value is less than 0.001.

First, we examined the chromosomal locations of the 27 Breg-related genes, as illustrated in **Figure 1A**. Next, we investigated the differential expression of these genes between normal lung tissues and LUAD tissues, revealing that the majority exhibited significant expression differences (**Figure 1B**). We then analyzed the SNV and CNV alterations of these 27 genes in LUAD. Somatic mutations in these genes were detected in 127 LUAD patients (22.8%), with missense mutations being the most prevalent. Among them, FLNA exhibited the highest mutation frequency (**Figure 1C**). Additionally, CNV analysis (**Figure 1D**) indicated that MYC had the highest frequency of copy number gain.

To further explore the immune landscape, we applied the ssGSEA algorithm to assess immune cell infiltration levels in LUAD patients. Moreover, the ESTIMATE algorithm was used to calculate StromalScore, ImmuneScore, and ESTIMATEScore. Correlation analysis demonstrated that Breg-related genes were significantly positively associated with most immune cell populations (**Figure 1E**) as well as immune-related scores (**Figure 1F**).

Finally, Gene Ontology (GO) analysis of these 27 genes revealed their involvement in multiple immune-suppressive biological processes, such as “negative regulation of immune system process,” “negative regulation of lymphocyte activation,” and “negative regulation of cytokine production” (**Figure 1G**).

### Consensus clustering identified two distinct Breg-related clusters

**Figure 2:**
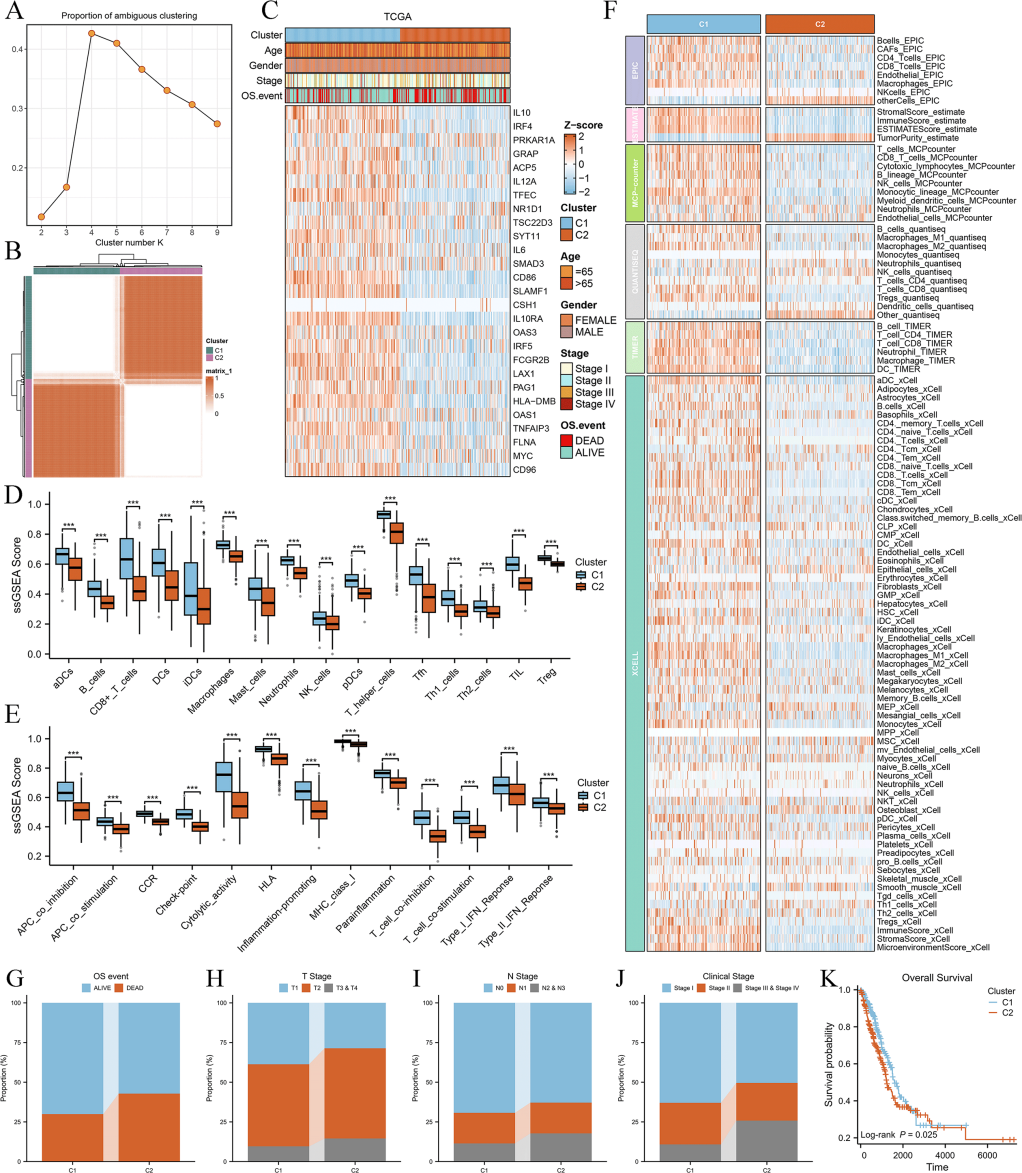
Consensus clustering of LUAD patients based on 27 Breg-related genes and their immune and clinical characteristics. **(A)** Heatmap of consensus clustering results for LUAD patients based on 27 Breg-related genes. **(B)** Proportion of Ambiguous Clustering (PAC) plot evaluating the clustering stability for different cluster numbers (k = 2–9). The PAC score, which quantifies clustering ambiguity, reaches its lowest value at k = 2, indicating that two clusters provide the most stable and optimal solution. **(C)** Expression patterns of the 27 Breg-related genes in the two identified clusters (C1 and C2). **(D, E)** Comparison of immune cell infiltration levels **(D)** and immune-related activities **(E)** between the two clusters based on ssGSEA analysis. **(F)** Immune cell infiltration levels in the two clusters evaluated using six distinct algorithms (EPIC, TIMER, MCPcounter, QuantiSeq, Xcell, and ESTIMATE, calculated in the IOBR package). **(G–J)** Distribution of OS event **(G)**, T stage **(H)**, N stage **(I)**, and clinical stage **(J)** in the two clusters. **(K)** Kaplan–Meier survival curves comparing overall survival between the two clusters. The log-rank test was used to assess statistical significance, revealing a significant difference in prognosis between C1 and C2. ‘***’ means P value is less than 0.001.

We performed molecular subtyping of LUAD patients based on 27 Breg-related genes. Patients were classified into k groups (k = 2–9), and the clustering heatmap (**Figure 2A**) and PAC plot (Proportion of Ambiguous Clustering, **Figure 2B**) indicated that k = 2 provided the optimal clustering solution. The expression patterns of these 27 genes in the two clusters are shown in **Figure 2C**, revealing that the majority of them were highly expressed in Cluster C1.

Next, we compared the immune characteristics between the two clusters. ssGSEA analysis demonstrated that all immune cell types (**Figure 2D**) and immune-related activities (**Figure 2E**) were significantly higher in C1 than in C2. Furthermore, six distinct algorithms (EPIC, ESTIMATE, TIMER, MCPcounter, QuantiSeq, and xCell) were employed to evaluate immune cell infiltration levels, showing that most immune cell populations exhibited higher infiltration in C1 (**Figure 2F**). These findings suggest that Cluster C1 exhibits a “hot immune” phenotype.

We then examined the relationship between the clusters and clinical characteristics of LUAD patients. Cluster C2 was significantly associated with unfavorable OS events (**Figure 2G**), more advanced T stage (**Figure 2H**), N stage (**Figure 2I**), and clinical stage (**Figure 2J**), as well as shorter overall survival (**Figure 2K**), indicating poorer clinical classification and prognosis. Therefore, patients in Cluster C2 exhibited a “cold immune” phenotype, which may contribute to disease progression.

### Immune-related molecular characteristics and immunogenicity differences between clusters

**Figure 3:**
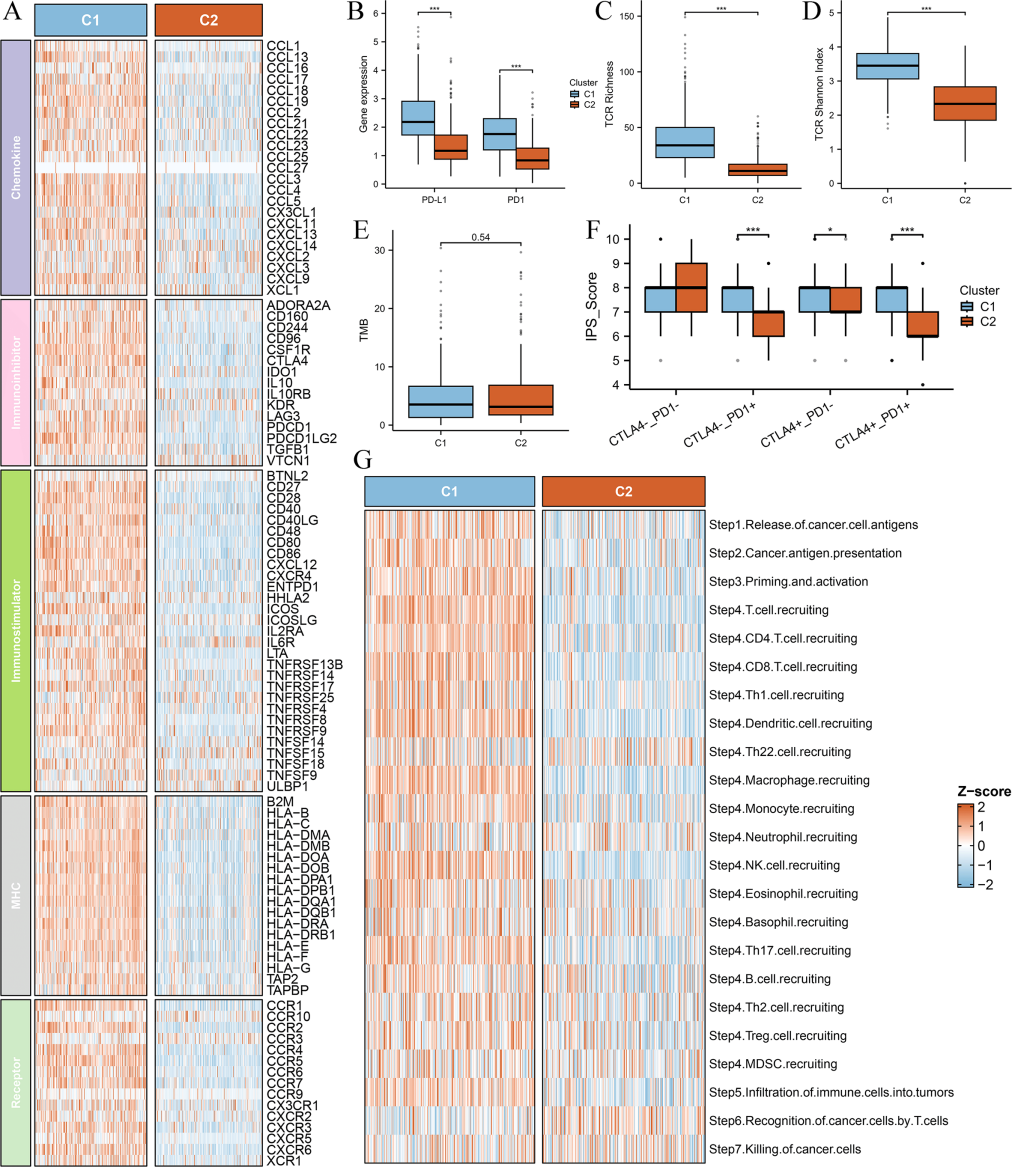
Comparison of immune-related molecular characteristics and immunogenicity between the two clusters. **(A)** Heatmap depicting the expression levels of immune-related molecules across the two clusters (C1 and C2). All analyzed immune-related genes show higher expression in Cluster C1 compared to Cluster C2, suggesting a more immunologically active tumor microenvironment in C1. The color gradient indicate differences in gene expression patterns. **(B)** Box plots comparing the expression levels of immune checkpoint molecules PD-1 (PDCD1) and PD-L1 (CD274) between the two clusters. **(C)** Box plot comparing T-cell receptor (TCR) richness between the two clusters. TCR richness quantifies the diversity of T-cell receptor clonotypes within a tumor, reflecting the potential for immune recognition and response. **(D)** Box plot comparing TCR Shannon diversity between the two clusters. The Shannon diversity index measures both the abundance and evenness of TCR clonotypes, providing insights into the heterogeneity of T-cell populations within the tumor. **(E)** Box plot comparing the tumor mutational burden (TMB) between the two clusters. TMB is a key biomarker for predicting response to immune checkpoint inhibitors, with higher values generally correlating with increased neoantigen presentation and potential immune recognition. **(F)** Box plot comparing the immunophenoscore (IPS) between the two clusters. The IPS score integrates multiple immune-related factors to assess the likelihood of response to immunotherapy, with higher scores indicating a more favorable immune landscape for immunotherapy efficacy. **(G)** Box plot comparing the anti-cancer immunity score between the two clusters. This score reflects the overall activation of immune-related pathways and cytotoxic activity within the tumor microenvironment. Differences between the clusters highlight variations in immune surveillance and potential therapeutic implications. ‘***’ means P value is less than 0.001.

To further investigate the immunological differences between the two Breg-related clusters, we first analyzed the expression of immune-related molecules. The heatmap (**Figure 3A**) demonstrated that all immune-related molecules were upregulated in Cluster C1 compared to Cluster C2. Specifically, PD-1 and PD-L1, two critical immune checkpoint molecules, were significantly higher in C1 (**Figure 3B**).

Next, we explored T-cell receptor (TCR) diversity between the clusters. TCR richness (**Figure 3C**) and TCR Shannon diversity (**Figure 3D**) were both significantly elevated in Cluster C1, indicating a more diverse and active TCR repertoire. Additionally, we assessed tumor mutational burden (TMB) between the clusters; however, no significant difference was observed (**Figure 3E**).

Furthermore, we evaluated the immunogenic potential of the clusters using the Immunophenoscore (IPS), a composite metric predictive of response to immune checkpoint inhibitors. IPS scores were significantly higher in Cluster C1 (**Figure 3F**), suggesting greater immune responsiveness in this subgroup. Finally, our investigation into cancer progression demonstrated that most essential steps, including cancer antigen presentation, priming and activation, exhibited increased activity in the C1 group (**Figure 3G**), reinforcing the notion that Cluster C1 possesses a more active immune microenvironment.

These findings further support the classification of Cluster C1 as a “hot immune” phenotype and Cluster C2 as a “cold immune” phenotype, highlighting their potential implications for immunotherapy responsiveness and disease progression.

### Differential analysis and single-cell RNA-sequencing analysis

**Figure 4:**
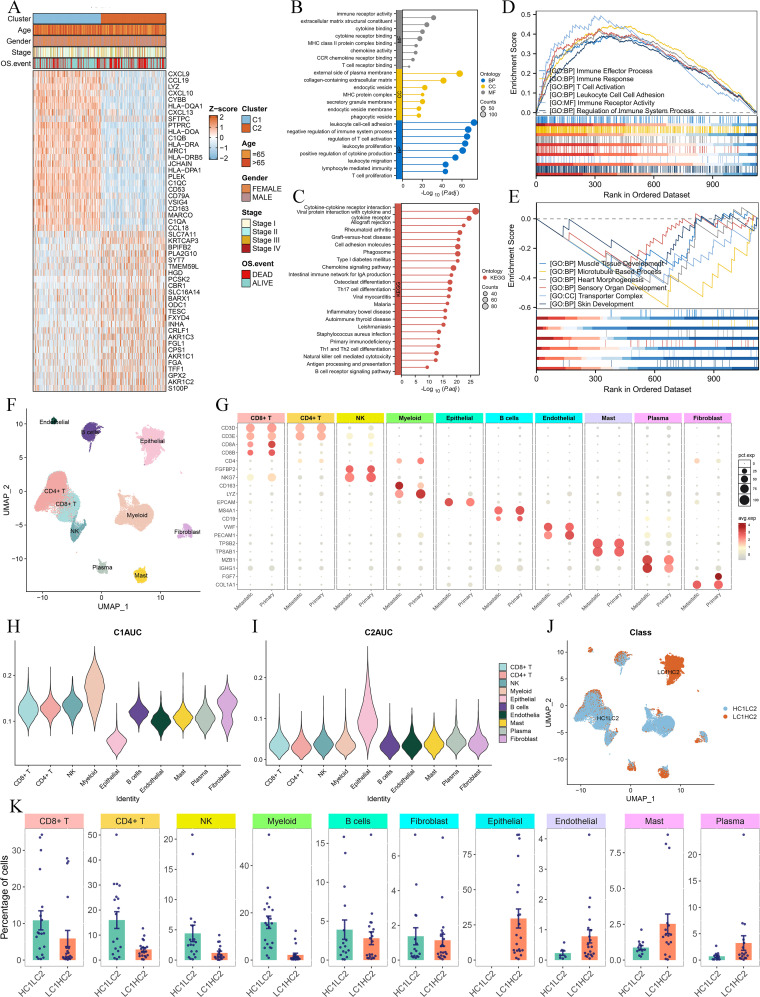
Differential gene expression and single-cell RNA-sequencing analysis of the two clusters. **(A)** Heatmap showing the top 50 DEGs between Cluster C1 and Cluster C2, with the top 25 upregulated genes in each cluster. DEGs were identified using the limma package, with the criteria of an adjusted P-value (Padj) < 0.001 and an absolute log2 fold change > 0.5. **(B, C)** GO **(B)** and KEGG **(C)** analysis. **(D, E)** GSEA analysis displaying the functions associated with gene sets highly expressed in C1 **(D)** and C2 **(E)**. **(F, G)** UMAP plot of single-cell RNA-sequencing data **(F)** and marker gene expression for each cell type **(G)**. **(H, I)** Violin plots of C1AUC **(H)** and C2AUC **(I)** gene set activity scores across cell types. **(J)** UMAP plot showing the distribution of cells in HC1LC2 and LC1HC2 groups, and **(K)** comparison of cell type composition between different groups.

To further elucidate the differences between the two Breg-related clusters, we performed a differential expression analysis. We defined the differentially expressed genes (DEGs) as those with an adjusted P-value (Padj) < 0.001 and an absolute |log2(Fold-change)| > 0.5 (**Supplementary Table S2**). The heatmap presents the top 50 DEGs, with the top 25 genes significantly upregulated in Cluster C1 and the top 25 genes significantly upregulated in Cluster C2 (**Figure 4A**). Next, GO analysis of these DEGs revealed their involvement in immune-related biological functions, including “immune receptor activity,” “T cell receptor binding,” and “negative regulation of immune system process” (**Figure 4B**). KEGG analysis further demonstrated that these genes were enriched in immune-related signaling pathways (**Figure 4C**). GSEA showed that genes highly expressed in Cluster C1 were associated with immune-related functions such as “immune effector process,” “immune response,” and “T cell activation” (**Figure 4D**), whereas genes highly expressed in Cluster C2 were enriched in biological processes such as “muscle tissue development,” “microtubule-based process,” and “heart morphogenesis” (**Figure 4E**).

To gain deeper insight into the cellular composition of the two clusters, we performed single-cell RNA sequencing (scRNA-seq) analysis using the GSE131907 dataset. The UMAP plot illustrates the clustering of single-cell transcriptomic data (**Figure 4F**), while **Figure 4G** shows the marker genes used to define each cell type. We then applied the AUCell algorithm to compute gene set activity scores based on the top 50 upregulated genes in Cluster C1 and Cluster C2, generating two scores: C1AUC and C2AUC. The violin plots depict the distribution of C1AUC (**Figure 4H**) and C2AUC (**Figure 4I**) across different cell types. Subsequently, we stratified all cells into four groups based on the median values of C1AUC and C2AUC: high-C1AUC (HC1), low-C1AUC (LC1), high-C2AUC (HC2), and low-C2AUC (LC2). We hypothesized that cells classified as high-C1AUC & low-C2AUC (HC1LC2) best represented the characteristics of Cluster C1, while those classified as low-C1AUC & high-C2AUC (LC1HC2) were most representative of Cluster C2. The UMAP distribution of HC1LC2 and LC1HC2 cells is shown in **Figure 4J**.

We then compared the cellular composition between HC1LC2 and LC1HC2 groups (**Figure 4K**). The results revealed that immune cells known to play critical roles in anti-tumor immunity, including CD4+ T cells, CD8+ T cells, B cells, myeloid cells, and NK cells, were more abundant in the HC1LC2 group. In contrast, mast cells, plasma cells, and endothelial cells were more prevalent in the LC1HC2 group. Notably, all epithelial (malignant) cells were exclusively enriched in the LC1HC2 group.

These findings further reinforce the notion that Cluster C1 exhibits a “hot immune” phenotype characterized by active anti-tumor immunity, whereas Cluster C2 displays a “cold immune” phenotype potentially facilitating tumor progression.

### BREGI construction and validation

To select key genes from the DEGs for constructing the Breg-related gene signature, we applied the following criteria:

1. Genes must be present in all eight selected datasets to ensure the model is applicable across different cohorts.
2. Genes must demonstrate prognostic significance in TCGA, GSE72094, and GSE68465 (P-value < 0.05 in univariate Cox regression analysis), as these datasets have the largest sample sizes and the highest statistical power.

**Figure 5:**
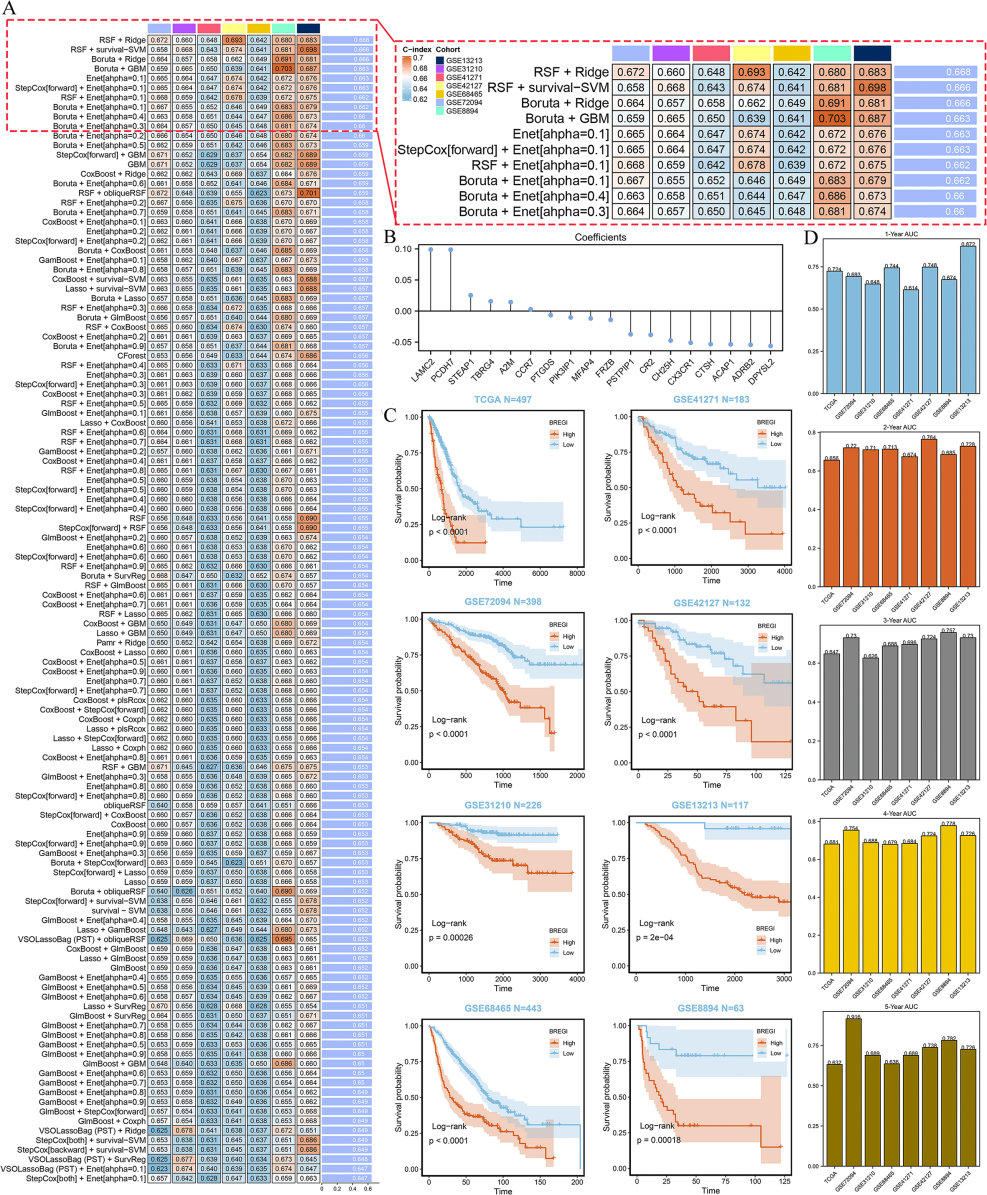
Construction and Validation of the BREGI. **(A)** Schematic representation of the BREGI construction process using an AI-driven approach. The heatmap displays the top 120 algorithmic combinations with the highest average C-index across seven GEO datasets. The combination of Random Survival Forest (RSF, ntree=10) and Ridge regression (10-fold cross-validation, nfold=10), which achieved the highest average C-index, was chosen for final model construction. **(B)** Coefficients assigned to each of the 18 genes composing BREGI, determined by the Ridge regression algorithm. The magnitude and direction of these coefficients indicate the contribution of each gene to the final BREGI. **(C)** Kaplan-Meier survival analysis comparing the prognosis of LUAD patients stratified into high- and low-BREGI groups based on an optimal threshold. Across all cohorts, the high-BREGI group exhibited significantly poorer prognosis, confirming the prognostic relevance of BREGI. Statistical significance was determined using the log-rank test. **(D)** Time-dependent receiver operating characteristic (ROC) curve analysis assessing the predictive accuracy of BREGI for 1-year, 3-year, and 5-year overall survival. The area under the curve (AUC) values across different time points indicate the strong predictive capability and robustness of BREGI as a prognostic biomarker for LUAD patients.

This process led to the identification of 32 genes for model construction. We then tested 274 algorithmic combinations on the TCGA dataset and calculated the C-index for each across the validation cohorts. The combination of RSF and ridge integration produced the highest average C-index of 0.668 across the validation cohorts. Based on this, we selected this combination to construct the Regulatory B Cell-related Index (BREGI, **Figure 5A**). The BREGI is composed of 18 Breg-related genes. The Ridge algorithm assigned a coefficient to each of these genes (**Figure 5B**, **Supplementary Table S2**). By multiplying the expression level of each gene by its respective coefficient and summing the results, the BREGI score is calculated. Furthermore, we analyzed the correlation between BREGI, the 18 genes composing BREGI, and clinical factors of LUAD patients (**Supplementary Figure S1A**). Higher BREGI, as well as elevated expression of LAMC2, PCDH7, STEAP1, and TBRG4, were associated with more aggressive clinical features. The differential expression of these 18 Breg-related genes between normal lung tissue and LUAD tissue is shown in **Supplementary Figure S1B**. Next, we examined the relationship between these 18 genes and immune cells and found significant correlations with various immune cell populations (**Supplementary Figure S1C**). GO analysis revealed that these 18 genes were involved in multiple immune-related functions, such as “negative regulation of immune effector process,” “negative regulation of complement activation,” and “negative regulation of leukocyte-mediated immunity” (**Supplementary Figure S1D**, indicating that these genes are significantly associated with Breg cells.

Then we stratified LUAD patients into high- and low-BREGI groups based on the optimal BREGI threshold. The high-BREGI group showed significantly worse prognosis compared to the low-BREGI group across all cohorts (**Figure 5C**). Furthermore, time-dependent ROC analysis revealed that BREGI demonstrated high AUC values when predicting patient prognosis over 1–5 years, confirming its predictive accuracy and reliability (**Figure 5D**). To test whether BREGI can serve as an independent prognostic factor for LUAD patients, we conducted both univariate (**Supplementary Figure S2A**) and multivariate (**Supplementary Figure S2B**) Cox regression analyses across all cohorts. Except for the multivariate Cox regression analysis in the GSE31210 cohort, where the p-value for BREGI was 0.069, BREGI demonstrated significant prognostic value in all other cohorts. These analyses indicate the robust predictive performance of BREGI and its potential as an independent prognostic factor for LUAD patients.

### Comparison of BREGI with clinical factors and published prognostic models

**Figure 6:**
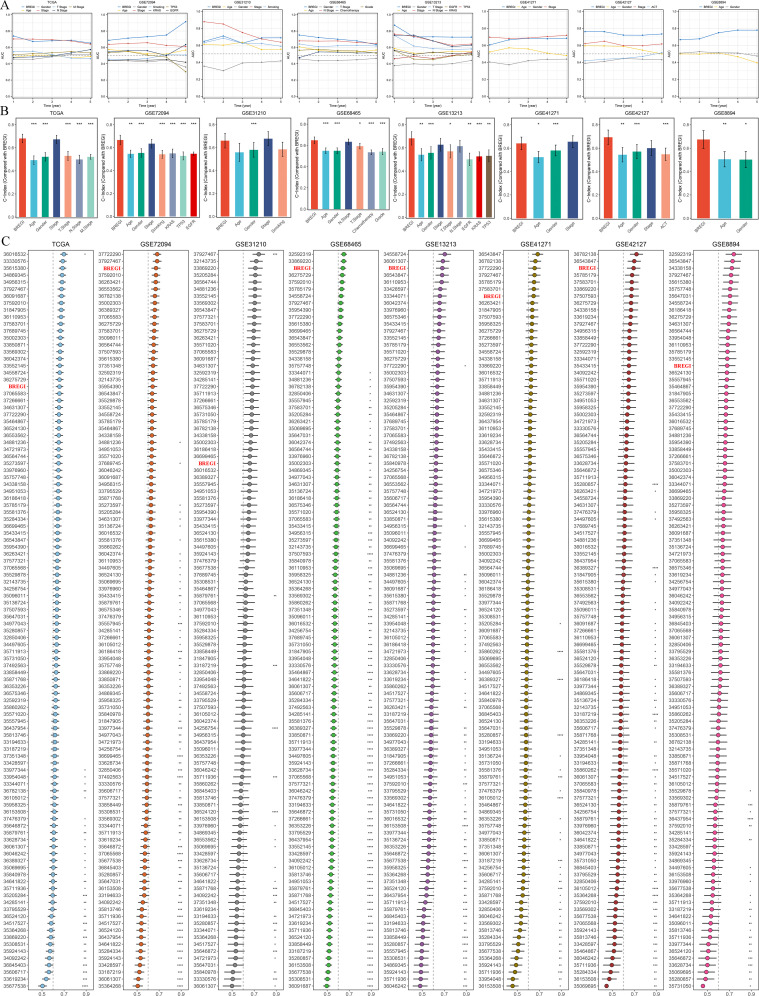
Comparative Performance of BREGI Against Clinical Features and Gene Signatures. **(A)** Comparison of the area under the curve (AUC) values between BREGI and clinical factors across eight independent cohorts, demonstrating BREGI’s superior discriminatory power in most cases. **(B)** Concordance index (C-index) comparison between BREGI and clinical variables, further supporting its prognostic accuracy across different datasets. **(C)** Evaluation of BREGI’s C-index performance relative to 105 published gene signatures across eight cohorts. ‘*’ means P value is less than 0.05, ‘**’ means P value is less than 0.01, ‘***’ means P value is less than 0.001.

To further demonstrate the superiority of BREGI, we compared it with clinical features. Specifically, we assessed the AUC values (**Figure 6a**) and concordance indices (C-index, **Figure 6b**) of BREGI and clinical factors across eight cohorts, revealing that BREGI outperformed most clinical variables. Furthermore, we compiled 105 previously published gene signatures associated with various phenotypes and compared their C-index with that of BREGI across the eight cohorts (**Figure 6c**). The results indicated that BREGI ranked relatively lower in the TCGA cohort, likely due to overfitting, as most studies used TCGA as a training set. However, in the validation cohorts, except for GSE31210, BREGI demonstrated superior C-index performance compared to most models. These findings further highlight the robustness and superiority of BREGI.

### Development and validation of the nomogram

Given the prognostic significance of age, gender, and clinical stage in LUAD patients, we proposed that incorporating these variables with BREGI could enhance predictive accuracy. To test this hypothesis, we constructed a nomogram integrating BREGI with age, gender, and clinical stage (**Supplementary Figure S3A**). In the TCGA cohort, this model achieved a C-Index of 0.715, demonstrating strong predictive performance. Further validation through calibration curves (**Supplementary Figure S3B**) and decision curve analysis (DCA) (**Supplementary Figure S3C**) affirmed its reliability in prognostic estimation. Notably, ROC analyses (**Supplementary Figures S3D-F**) indicated that the nomogram outperformed individual predictors, including BREGI, age, gender, and clinical stage, in forecasting patient outcomes. These findings highlight the potential of this integrated nomogram as a valuable tool for clinical decision-making and risk stratification in LUAD.

### Association between BREGI and the tumor immune microenvironment

**Figure 7:**
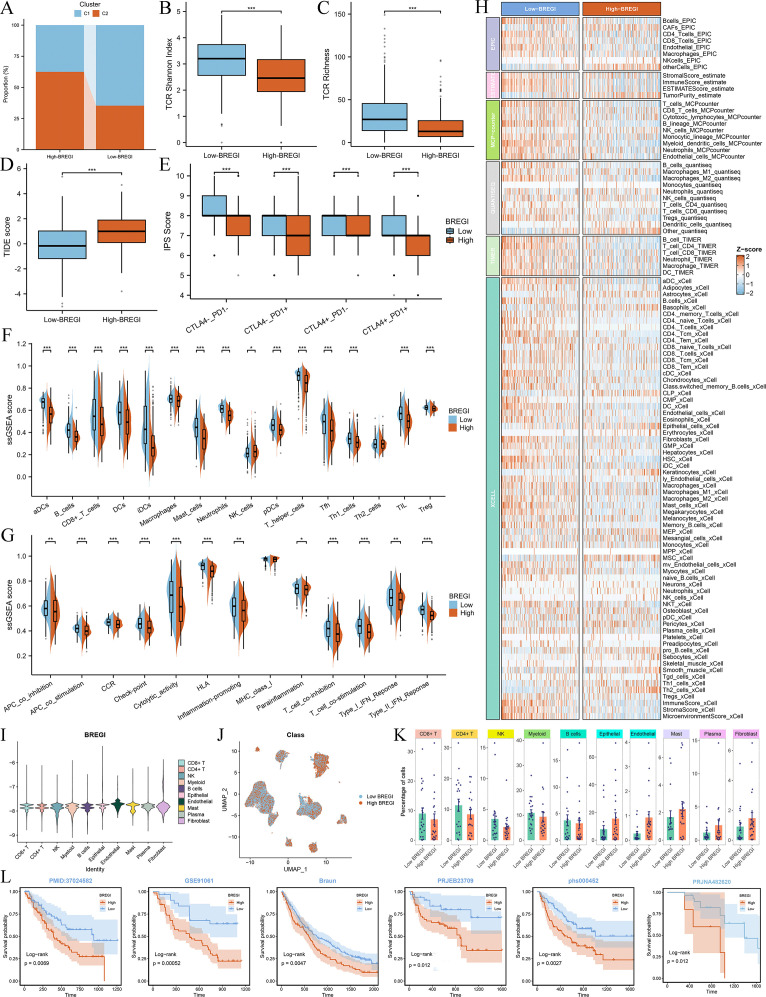
Association between BREGI and TME features. **(A)** Proportion of Cluster C1 and C2 patients in low- and high-BREGI groups. **(B, C)** Comparison of TCR Shannon diversity **(B)** and TCR richness **(C)** between BREGI subgroups. **(D, E)** Relationship between BREGI and TIDE **(D)** and IPS **(E)** scores. **(F)** Immune cell infiltration levels and **(G)** immune-related activities in BREGI subgroups via ssGSEA. **(H)** Comparison of immune cell infiltration using six computational algorithms. **(I)** Single-cell BREGI expression distribution. **(J)** Stratification of single-cell populations into high- and low-BREGI groups. **(K)** Proportion of different cells in BREGI subgroups. **(L)** Prognostic comparison of immunotherapy-treated patients between BREGI subgroups in six real-world cohorts. ‘*’ means P value is less than 0.05, ‘**’ means P value is less than 0.01, ‘***’ means P value is less than 0.001.

To further explore the relationship between BREGI and the TME, we first examined its association with Breg-related clusters. Our analysis revealed that patients in Cluster C1 exhibited a “hot immune” phenotype. Notably, the proportion of Cluster C1 patients was significantly higher in the low-BREGI group than in the high-BREGI group, suggesting a stronger association between low BREGI and the hot immune phenotype (**Figure 7A**). ssGSEA analysis further demonstrated that the majority of immune cell infiltration levels (**Figure 7F**) and immune-related activities (**Figure 7G**) were elevated in the low-BREGI group. Using six algorithms, we consistently observed higher immune cell infiltration in the low-BREGI group (**Figure 7H**). Correlation analysis showed that BREGI was negatively associated with most immune cells (**Supplementary Figure S4C**) but positively correlated with other TME components, such as keratinocytes and epithelial cells.

To validate these findings at the single-cell level, we calculated BREGI in the GSE131907 scRNA-seq dataset (**Figure 7I**) and stratified all cells into high- and low-BREGI groups based on the median BREGI value (**Figure 7J**). The low-BREGI group exhibited a higher proportion of CD8+ T cells, CD4+ T cells, B cells, NK cells, and myeloid cells, whereas epithelial and endothelial cell proportions were lower (**Figure 7K**), resembling the HC1LC2 group (**Figure 4K**). Based on these findings, we hypothesize that similar to Cluster C1, the low-BREGI group exhibits a “hot immune” phenotype, while the high-BREGI group is characterized by a “cold immune” profile.

To assess the relevance of BREGI to immunotherapy, we analyzed its association with TCR Shannon diversity (**Figure 7B**), TCR richness (**Figure 7C**), TIDE score (**Figure 7D**), and IPS score (**Figure 7E**). The low-BREGI group exhibited significantly higher TCR Shannon diversity, TCR richness, and IPS scores, indicating greater sensitivity to immunotherapy. Conversely, the high-BREGI group had higher TIDE scores, which predict resistance to immunotherapy, further supporting the link between low BREGI expression and improved immunotherapy response.

Additionally, we compared the expression of various immune-related molecules between the BREGI subgroups (**Supplementary Figure S4A**) and found that most were upregulated in the low-BREGI group, with BREGI expression negatively correlated with their levels (**Supplementary Figure S4B**). Finally, we calculated BREGI expression in six real-world immunotherapy cohorts and assessed patient outcomes following immune checkpoint blockade. The results demonstrated significantly worse prognosis in high-BREGI patients, suggesting that low-BREGI patients derive greater clinical benefit from immunotherapy (**Figure 7L**).

### Genomic variations and their relationship with BREGI status

Furthermore, we performed a comprehensive genomic comparison between the BREGI-high and BREGI-low groups within the TCGA dataset. Initially, we focused on the top 20 genes with the highest mutation frequencies. Our analysis revealed that patients in the BREGI-high group exhibited a significantly higher overall mutation frequency (96.3%, **Supplementary Figure S5A**) compared to those in the BREGI-low group (90.53%, **Supplementary Figure S5B**). Further analysis demonstrated that the top 30 genes with the most pronounced differences in mutation frequency were consistently more frequently mutated in the BREGI-high group (**Supplementary Figure S5C**), with significant co-mutation relationships observed among these genes (**Supplementary Figure S5D**).

Moreover, BREGI expression was found to be significantly positively correlated with multiple genomic features, including silent mutations, non-silent mutations, aneuploidy score, fraction of altered genome (FGA), fraction of genome gained (FGG), and fraction of genome lost (FGL) (**Supplementary Figure S5E**). Additionally, our findings revealed substantial differences in CNV events between the two groups. Patients in the BREGI-high group exhibited heightened genomic instability, characterized by a greater frequency and complexity of CNV events (**Supplementary Figure S5F**), whereas those in the BREGI-low group displayed lower levels of genomic instability (**Supplementary Figure S5G**). ChromPlots further demonstrated that patients in the BREGI-high group had significantly higher G-scores (**Supplementary Figure S5H**) compared to those in the BREGI-low group (**Supplementary Figure S5I**).

These findings suggest that LUAD patients with high BREGI expression are more likely to exhibit aggressive and malignant genomic characteristics.

### Identification of potential therapeutic agents for high-BREGI patients

Given the significantly worse prognosis, increased malignancy, and immunotherapy resistance observed in high-BREGI patients, there is an urgent need to identify potential therapeutic options targeting this subgroup. To address this, we compared the IC50 values of various drugs between the high- and low-BREGI groups, where lower IC50 values indicate higher sensitivity. The results revealed that multiple chemotherapeutic agents, including paclitaxel, docetaxel, and vinorelbine, as well as targeted therapies such as gefitinib and cetuximab, exhibited lower IC50 values in the high-BREGI group (**Supplementary Figure S6A**). Furthermore, correlation analysis demonstrated a significant negative association between BREGI expression and the IC50 values of these drugs (**Supplementary Figure S6B**), suggesting that they may serve as potential treatment options for high-BREGI patients.

### Knockdown of TBRG4 inhibited LUAD cells’ proliferation, migration, and invasion

**Figure 8:**
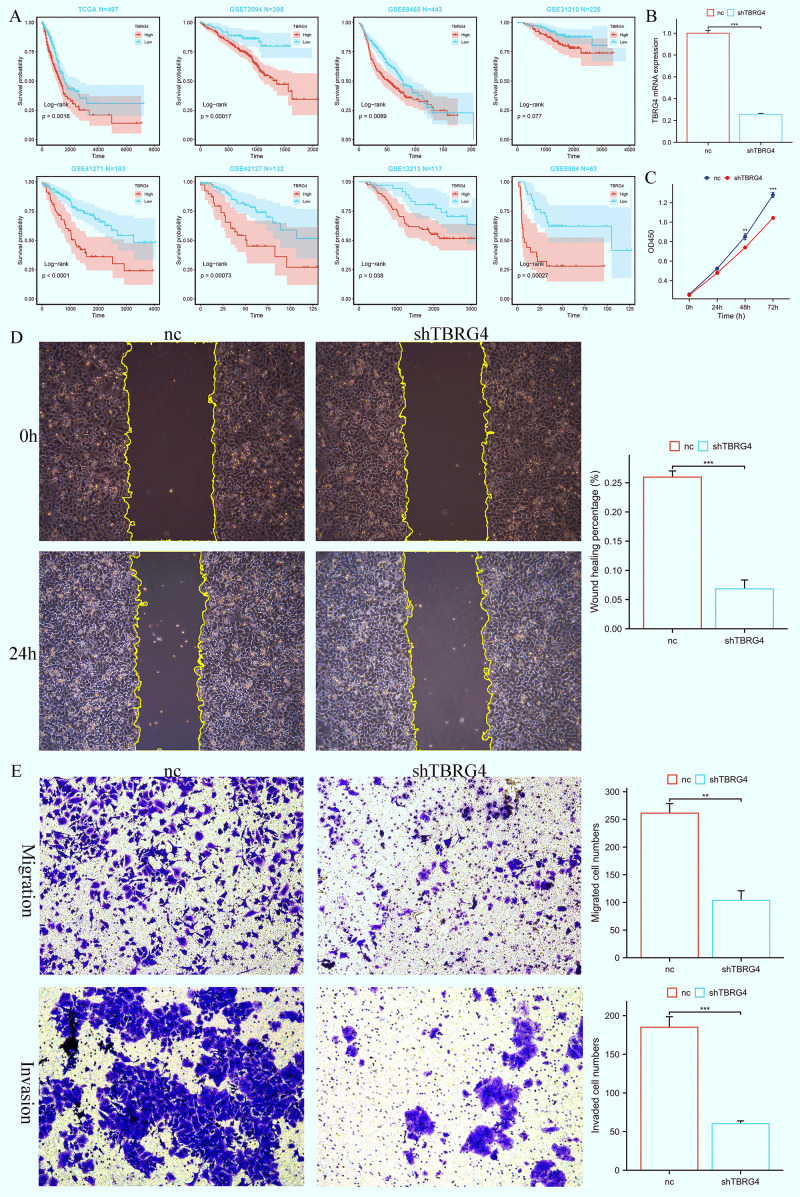
TBRG4 correlates with poor prognosis in LUAD and its knockdown inhibits malignant phenotypes of LUAD cells. **(A)** Survival analysis showing the prognostic significance of TBRG4 in LUAD. **(B)** qRT-PCR analysis verifying efficient knockdown of TBRG4 in A549 cells transfected with sh-TBRG4. **(C)** CCK-8 assay demonstrating reduced proliferation capacity of A549 cells after TBRG4 knockdown. **(D)** Wound healing assay revealing impaired migration of A549 cells after TBRG4 knockdown. **(E)** Transwell assays showing suppressed migration and invasion of A549 cells upon TBRG4 knockdown. ‘**’ means P value is less than 0.01, ‘***’ means P value is less than 0.001.

Among the 18 genes incorporated in the construction of BREGI, the role of TBRG4 in lung adenocarcinoma remained relatively less unexplored., prompting us to further investigate its functional role. Bioinformatics analyses first identified TBRG4 as a significant risk factor, with elevated TBRG4 expression correlating with poorer prognosis in LUAD patients (**Figure 8A**). To validate its biological relevance, we subsequently performed *in vitro* experiments to dissect the potential phenotypic roles of TBRG4 in LUAD progression. Efficient knockdown of TBRG4 in human LUAD A549 cells was achieved using shRNA, as confirmed by significant reduction in TBRG4 mRNA expression (**Figure 8B**). The CCK-8 assays revealed that TBRG4 depletion exerted a profound inhibitory effect on A549 cell proliferation (**Figure 8C**). Consistently, wound healing assays demonstrated impaired migratory capacity in TBRG4-knockdown A549 cells (**Figure 8D**). Furthermore, transwell assays validated that TBRG4 silencing suppressed both migratory and invasive properties of A549 cells (**Figure 8E**).

Collectively, these findings establish that TBRG4 knockdown robustly attenuates the malignant phenotypes of LUAD cells, including proliferation, migration, and invasion. This functional evidence, coupled with its identification as a risk factor via bioinformatics, underscores TBRG4’s pathogenic relevance in LUAD progression.

## Discussion

Lung adenocarcinoma (LUAD) continues to be the leading cause of cancer-related mortality across all cancer types, representing a significant and ongoing threat to global public health (38). Previous studies have demonstrated that the initiation and progression of LUAD are driven by intricate biological processes, including a wide array of genetic alterations and epigenetic modifications (39, 40). Various staging systems have been developed and applied in clinical practice to assess patient prognosis. However, these models primarily focus on clinicopathological characteristics, overlooking the pivotal role of intricate molecular mechanisms in the initiation and progression of LUAD (41, 42). As a consequence, advancements in patient outcomes have remained minimal. Therefore, the discovery of more effective predictive biomarkers for treatment response and prognosis could play a crucial role in refining personalized therapeutic approaches and enhancing prognostic management for individuals diagnosed with LUAD.

Effector T cells, recognized for their pivotal role in directly targeting and eliminating tumor cells, have been a central focus in tumor immunology research. However, T cell-mediated effector mechanisms alone are inadequate to counteract the adaptive and evasive strategies employed by malignant cancers (43). Researchers are increasingly focusing on uncovering the mechanisms that enable cancer cells to evade the attack of effector T cells. This has led to the identification of a specialized subset of B cells, known as regulatory B cells (Bregs), which exhibit strong immunosuppressive properties and play a crucial role in dampening immune surveillance in cancer (44). Although Bregs have been implicated in various cancers, their precise functions and prognostic significance in LUAD remain inadequately explored. This study first provides a comprehensive analysis of the role of Breg-related genes in LUAD, identifying novel Breg-related clusters and the Breg-related Index (BREGI), all of which have a significant impact on the tumor microenvironment (TME) of LUAD. Our clustering analysis revealed that LUAD patients could be stratified into distinct immune subtypes based on 27 Breg-related genes, with Cluster C1 exhibiting a “hot immune” phenotype characterized by increased immune cell infiltration and active anti-tumor immune responses. Moreover, BREGI serves as a robust prognostic tool for LUAD patients, outperforming traditional clinical factors and several previously published gene signatures in terms of predictive accuracy. Interestingly, we observed a significantly higher proportion of Cluster C1 patients in the low-BREGI group, whereas the high-BREGI group was predominantly composed of Cluster C2 patients, who displayed a more immunosuppressive “cold immune” phenotype. These findings suggest that elevated BREGI is closely linked to an immune-excluded or immune-suppressive tumor microenvironment, which may contribute to immune evasion and tumor progression. The single-cell RNA sequencing (scRNA-seq) analysis further validated these observations by demonstrating significant differences in immune cell composition between high- and low-BREGI groups at the cellular level. The low-BREGI group exhibited higher proportions of CD8+ T cells, CD4+ T cells, B cells, NK cells, and myeloid cells—hallmarks of an immune-active environment. In contrast, epithelial and endothelial cells were more abundant in the high-BREGI group, aligning with an immune-cold, tumor-promoting phenotype. Additionally, our study highlights the clinical relevance of BREGI in predicting response to immunotherapy. Previous research has established that the T-cell receptor (TCR) plays a critical role in recognizing antigens presented by the major histocompatibility complex (MHC). Furthermore, analyzing the TCR repertoire has emerged as a valuable biomarker for patient stratification and monitoring, particularly in the context of immunotherapy (45–47). The low-BREGI group exhibited significantly higher TCR Shannon diversity and TCR richness, suggesting a more diverse and active T-cell receptor repertoire, which is commonly associated with better immunotherapy outcomes. Furthermore, the low-BREGI group had higher IPS scores and lower TIDE scores, indicating enhanced sensitivity to immune checkpoint blockade therapies. These results were further supported by real-world immunotherapy cohorts, where high-BREGI patients had significantly worse prognosis compared to low-BREGI patients, reinforcing the potential of BREGI as a biomarker for immunotherapy response prediction.

Regulatory T cells (Tregs) and regulatory B cells (Bregs) are two distinct immune cell subsets known for their potent immunosuppressive properties. These cells play a critical role in modulating the immune microenvironment by suppressing the activation and function of effector immune cells, thereby maintaining immune tolerance and preventing excessive inflammatory responses. The systematic analysis of Treg-related genes in cancer has been extensively explored in previous studies. Some studies have comprehensively examined the role of Treg-associated genes in various malignancies, including pancreatic cancer (48), clear cell renal cell carcinoma (49, 50), breast cancer (51), colorectal cancer (52), esophageal squamous cell carcinoma (53), and lung adenocarcinoma (54). However, the role of Breg-related genes in cancer has been scarcely studied, with Zhou et al. being the only researchers to systematically analyze Breg-associated genes in bladder cancer (19). Therefore, a systematic analysis of the role of Breg-related genes across different cancers is of significant importance. This study is the first to comprehensively investigate the function of Breg-associated genes in LUAD, filling a crucial gap in research on their involvement in this malignancy.

Some previously published studies have utilized traditional regression or machine learning techniques to construct prognostic models for lung adenocarcinoma based on phenotype-associated genes (55–57). The integration of multiple machine learning approaches offers a more effective strategy for developing models with enhanced predictive performance. Inspired by these studies, we developed an advanced artificial intelligence (AI) framework that integrates 32 algorithms spanning traditional regression, machine learning, and deep learning. By systematically combining these algorithms, we generated a total of 274 unique model configurations. To mitigate overfitting—where a model performs well on the training set but exhibits significantly reduced predictive power in validation cohorts—we selected the combination with the highest average C-Index across eight independent validation datasets. The resulting model was designated as ‘BREGI.’ This framework represents an upgraded version of our previous research. In our earlier studies, we employed a similar approach to construct LUAD prognostic models related to dendritic cells (DC). At that time, we utilized only 10 algorithms, generating just 91 different model combinations to identify the optimal predictive model (58). In this study, we expanded the algorithm pool to 32 and increased the number of model combinations to 274, significantly enhancing the flexibility and robustness of model selection. This advancement not only strengthens the predictive capabilities of our framework but also provides valuable insights and a solid reference for future prognostic model development.

BREGI is composed of 18 genes (LAMC2, PCDH7, STEAP1, TBRG4, A2M, CCR7, PTGDS, PIK3IP1, MFAP4, FRZB, PSTPIP1, CR2, CH25H, CX3CR1, CTSH, ACAP1, ADRB2, DPYSL2). Many of these genes have been extensively studied and proven to be closely associated with the initiation and progression of LUAD. LAMC2 and STEAP1 been demonstrated to enhance the migration and invasion of lung adenocarcinoma cells, concomitant with the induction of epithelial-mesenchymal transition (EMT) (59, 60). Similarly, PCDH7 has been shown to play a pivotal role in LUAD progression. Both *in vitro* and *in vivo* experiments have confirmed that PCDH7 promotes LUAD growth and enhances resistance to anoikis. Notably, its overexpression significantly elevates intracellular triglyceride levels and upregulates the expression of FASN and ACC1 proteins, thereby suppressing anoikis in LUAD cells (61). Interestingly, LAMC2, STEAP1, and PCDH1 are all risk factors in our study. Interestingly, LAMC2, STEAP1, and PCDH1 have all been identified as risk factors in our study. We also observed that one of the genes comprising BREGI, CTSH, had been previously demonstrated in our earlier research to inhibit LUAD cell migration and invasion while promoting apoptosis (58). In our previous study, we constructed a prognostic model using dendritic cell (DC) markers, where CTSH was identified as one of the DC markers. In the present study, CTSH was identified as a Breg-associated gene, suggesting a potential link between Bregs and DCs.

Additionally, MFAP4 and ACAP1, two protective factors identified in this study, have been demonstrated to suppress the malignant phenotype of LUAD by upregulating their expression, thereby improving prognosis (62–64). Interestingly, CCR7 was identified in this study as a Breg-related gene and contributes to the construction of BREGI. However, its coefficient is notably small (0.002), indicating a relatively low weight in the model. The role of CCR7 in LUAD remains controversial. Some studies suggest that CCR7 knockout suppresses the malignant phenotype of LUAD cells (65), while others propose that high expression of CCR7 is associated with improved postoperative prognosis in LUAD patients (66). Furthermore, bioinformatics analyses have explored the roles of CH25H (67) and DPYSL2 (68) in LUAD; however, there is a lack of relevant *in vitro* or *in vivo* experiments to validate their specific functions. The exploration of the roles of the remaining genes in LUAD remains relatively limited. Therefore, focusing on these genes has the potential to address gaps in the field and provide valuable insights.

TBRG4 (Transforming Growth Factor Beta Regulator 4), one of the 18 genes comprising the BREGI, is involved in the progression of multiple cancers. Functional studies demonstrate that TBRG4 knockdown inhibits osteosarcoma cell proliferation, clonogenic ability, and invasion, while inducing apoptosis *in vitro*; *in vivo*, it suppresses tumor growth and metastasis (69). Furthermore, Tao et al. utilized transwell, wound healing, and CCK8 assays to demonstrate that knocking down TBRG4 significantly inhibits the migration, invasion, and proliferation of hepatocellular carcinoma (HCC) cells (70). Furthermore, its role in pancreatic cancer cells is similar, as knocking down TBRG4 has been shown to inhibit the migration, invasion, and epithelial-mesenchymal transition (EMT) of pancreatic cancer (71). However, previous studies have yielded limited insights into its role in the pathogenesis of LUAD. Given this significant knowledge gap, we prioritized functional characterization of TBRG4 to elucidate its potential involvement in LUAD progression. Inspired by the aforementioned studies, we set out to explore whether TBRG4 exerts an impact on the malignant phenotypes of LUAD. To address this, we employed CCK-8, transwell, and wound healing assays to systematically compare the migration, invasion, and proliferation capabilities between negative control (NC) cells and TBRG4 knockdown cells. The results of these experiments revealed that, in the TBRG4 knockdown group, all the aforementioned malignant phenotypes were significantly inhibited. This finding indicates that, similar to its role in the aforementioned cancers (including osteosarcoma, pancreatic cancer, and hepatocellular carcinoma), TBRG4 also modulates the malignant phenotypes of LUAD cells, thereby contributing to the progression of LUAD. This is theoretically and translationally significant. Demonstrating TBRG4 drives LUAD progression via regulating proliferation, migration, and invasion expands understanding of LUAD pathogenesis and identifies it as a potential therapeutic target. Given advanced LUAD’s high mortality and limited treatments, uncovering such roles of understudied BREGI components like TBRG4 aids in developing novel targeted strategies to improve prognosis. Moreover, TBRG4’s consistent pro-tumorigenic role across osteosarcoma, pancreatic cancer, HCC, and now LUAD suggests it is a conserved oncogenic factor, warranting broader oncology research, and further validates the robustness of BREGI as a functionally coherent gene set.

In this study, the Breg-Related Gene Index (BREGI) demonstrated robust predictive efficacy for patient prognosis across both training and validation cohorts. The exceptional predictive performance of BREGI prompted us to explore its potential underlying mechanisms. To this end, we employed multiple algorithms to compare the levels of immune cell infiltration between high-BREGI and low-BREGI groups. Our analysis revealed that tumors with high BREGI exhibited significantly reduced immune cell infiltration, indicative of a “cold tumor” phenotype (72) characterized by diminished anti-tumor immune activity. This attenuated immune infiltration likely facilitates tumor immune evasion and promotes disease progression, providing a plausible explanation for the markedly poorer survival outcomes observed in LUAD patients with elevated BREGI. The observed disparities in immune cell infiltration and immune activity between the high- and low-BREGI groups led us to investigate the potential utility of BREGI in predicting responses to immunotherapy. To this end, we first examined the relationship between BREGI and established biomarkers of immunotherapy response, including TCR richness, TCR Shannon index, TIDE score, and immunophenoscore (IPS). The results consistently indicated that patients in the low-BREGI group were more likely to benefit from immunotherapy. However, the efficacy of immunotherapy is influenced by multiple factors, including tumor immunogenicity, the abundance and functionality of tumor-infiltrating T cells, and the expression levels of immune checkpoint molecules. Therefore, validation in real-world immunotherapy cohorts was essential to confirm the predictive value of BREGI. To address this, we analyzed six independent immunotherapy cohorts, calculating BREGI scores for each. The results uniformly demonstrated that patients with high-BREGI scores who received immunotherapy had significantly worse prognoses compared to those with low-BREGI scores, further supporting the conclusion that low-BREGI patients derive greater benefit from immunotherapy. In summary, low-BREGI patients exhibit a higher likelihood of responding favorably to immunotherapy. With additional validation, BREGI has the potential to serve as a reliable biomarker for predicting immunotherapy response, thereby aiding in the stratification of patients for personalized treatment strategies.

Despite the utilization of robust open-source data to delineate two distinct characteristics of LUAD Breg-related subtypes and to establish a reliable AI-driven BREGI evaluation model, this study is constrained by several limitations. Firstly, the research is based on patient data derived from publicly available retrospective cohorts, lacking prospective real-world data necessary to validate the clinical applicability of the proposed scoring system. Second, while we observed that TBRG4 knockdown inhibits malignant phenotypes of LUAD cells, the underlying molecular mechanisms remain underexplored. Specifically, we have not identified the downstream effector molecules or signaling pathways through which TBRG4 exerts its regulatory effects. Additionally, due to financial and resource constraints, we were unable to conduct *in vivo* experiments to explore the functional roles of TBRG4 in LUAD. While our *in vitro* data robustly support TBRG4’s oncogenic role, further validation in animal models and clinical cohorts is warranted. Further experimental and investigative efforts are essential to achieve a more comprehensive understanding of the underlying molecular mechanisms. Nevertheless, we believe that this study represents the first systematic analysis of the role of Breg-related genes in LUAD, thereby holding significant value. It provides a foundational framework and insights for future research focused on Bregs. Moving forward, our research aims to validate the utility of BREGI in larger cohorts and to more systematically investigate the roles of BREGI-associated genes in LUAD.

## Data Availability

The datasets presented in this study can be found in online repositories. The names of the repository/repositories and accession number(s) can be found in the article/**Supplementary Material**.
