# Supplementary material for: Comprehensive analysis of regulatory B Cell related genes in prognosis and therapeutic response in lung adenocarcinoma
Source: Front Immunol. 2025 Jul 30;16:1595408. doi: 10.3389/fimmu.2025.1595408 (PMC12343544; doi:10.3389/fimmu.2025.1595408)
Supplement: Supplementary file 1 [file DataSheet1.docx]

**
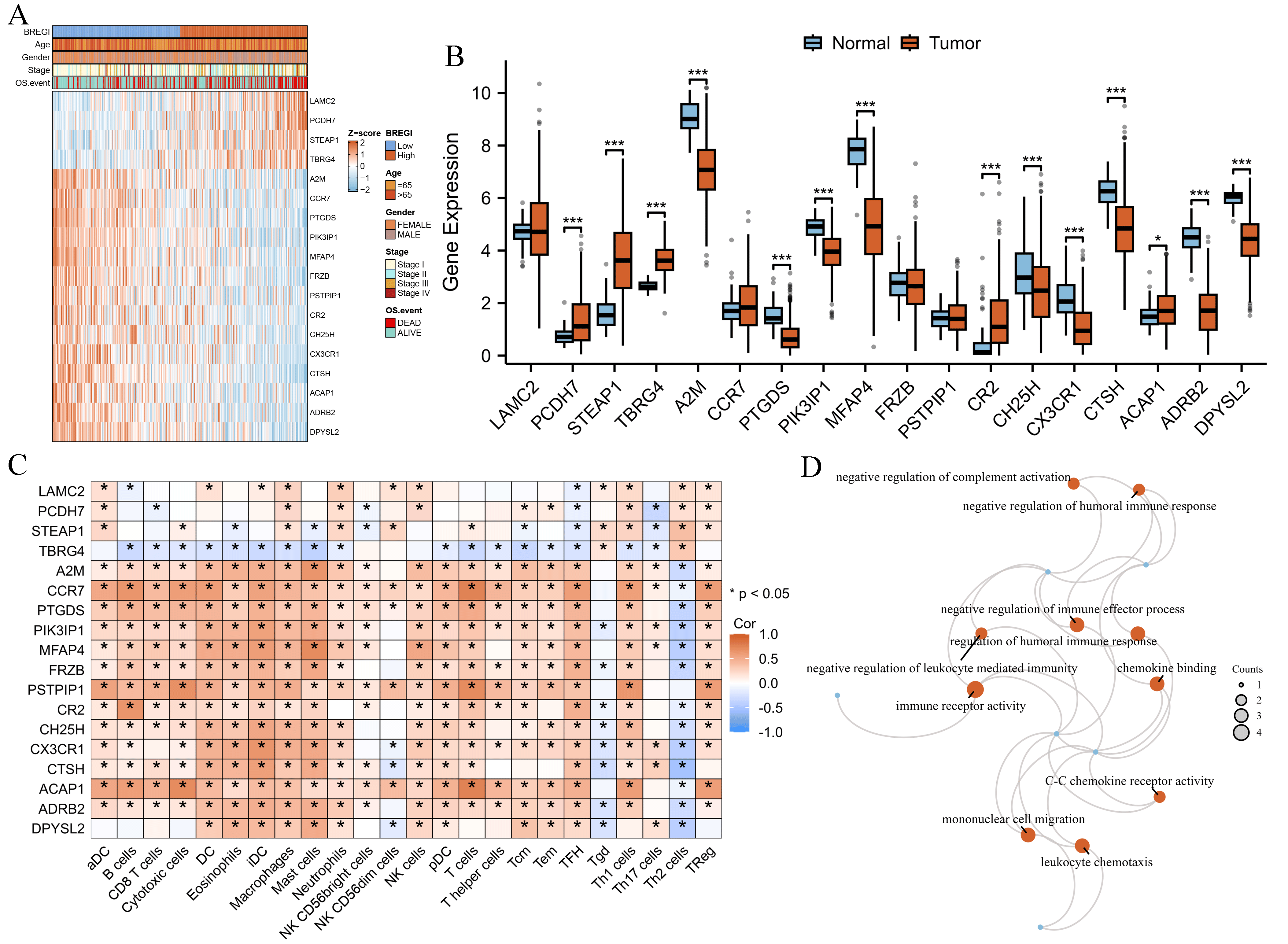
**

**Figure S1. Analysis of BREGI-related genes and their associations with clinical features and immune cells in LUAD.**

(A) Correlation between BREGI, the 18 BREGI-related genes, and clinical factors. (B) Differential expression of the 18 BREGI-related genes between normal lung tissue and LUAD tissue. (C) Correlation between the 18 BREGI-related genes and immune cell populations. (D) GO analysis of the 18 BREGI-related genes.

**
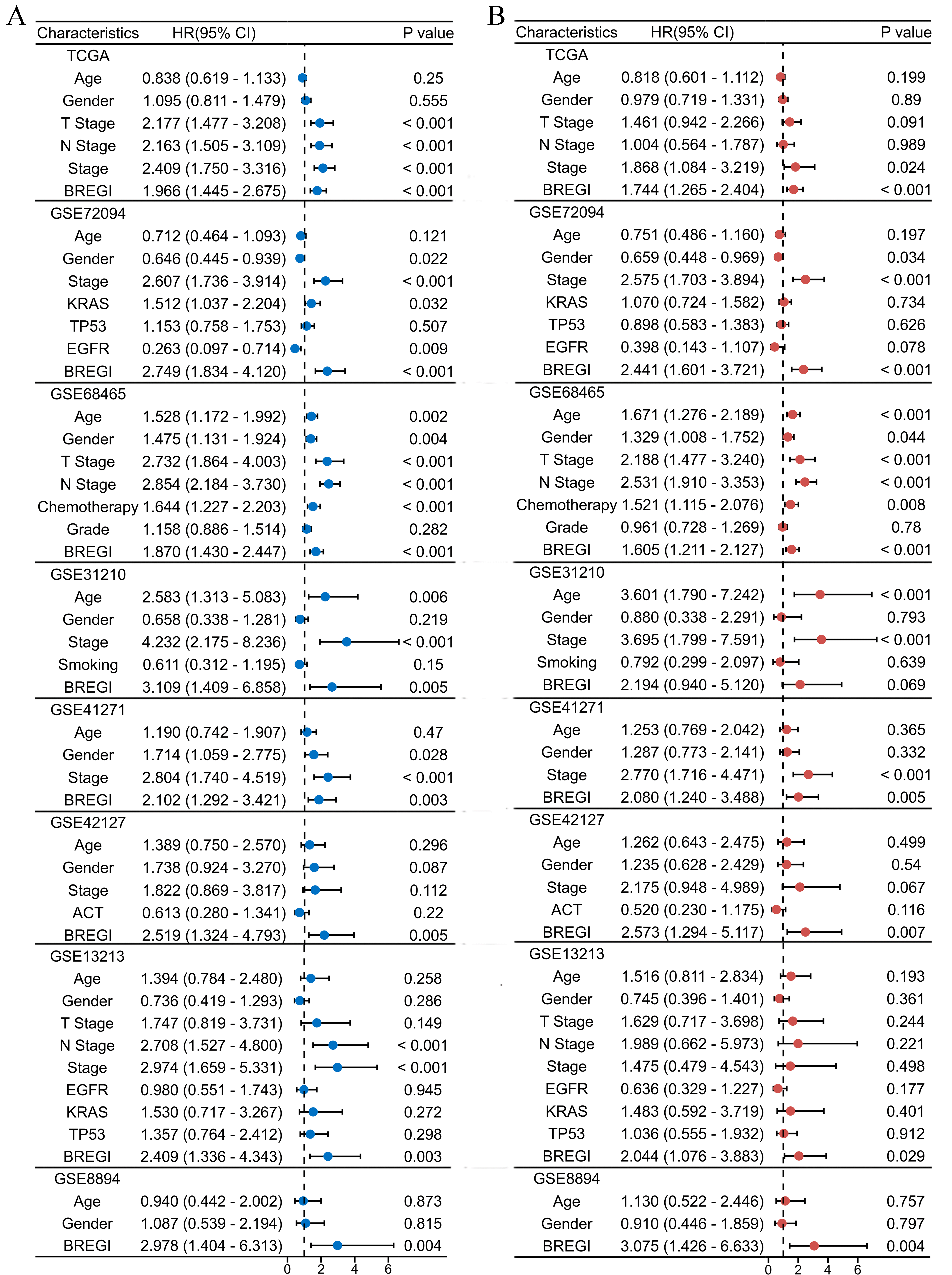
**

**Figure S2. Independent prognostic value of BREGI in LUAD.**

(A) Univariate Cox regression analysis of BREGI as a prognostic factor across all cohorts. (B) Multivariate Cox regression analysis of BREGI as an independent prognostic factor for LUAD patients in all cohorts, with the exception of the GSE31210 cohort, where the p-value was 0.069.


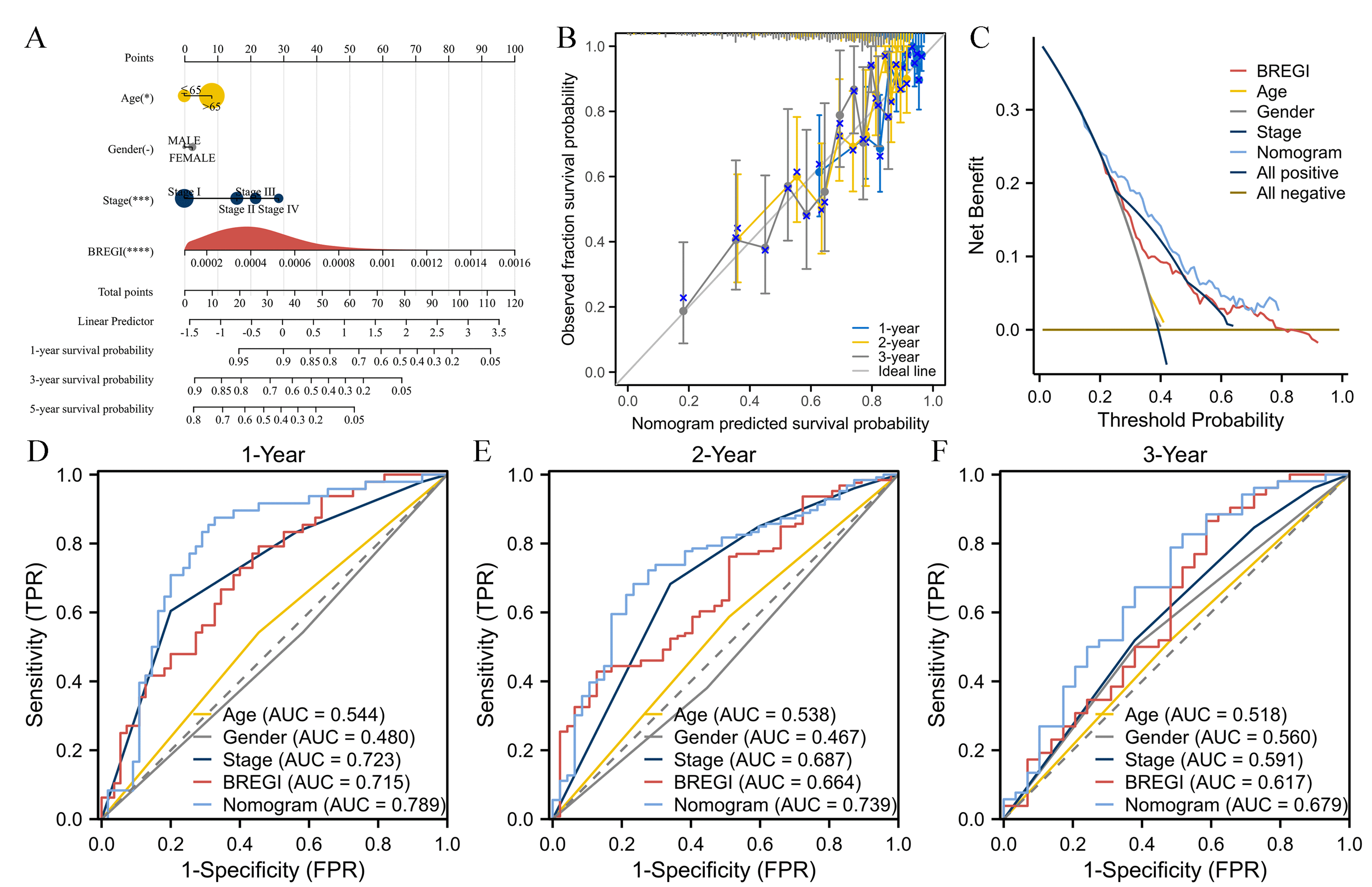


**Figure S3. Development and validation of the nomogram integrating BREGI and clinical factors.**

1. Nomogram incorporating BREGI, age, gender, and clinical stage. (B) Calibration curve assessing the nomogram’s predictive accuracy. (C) Decision curve analysis (DCA) evaluating its clinical utility. (D-F) ROC curves comparing the nomogram’s performance with individual predictors for 1- (D), 2- (E), and 3- (F) years.


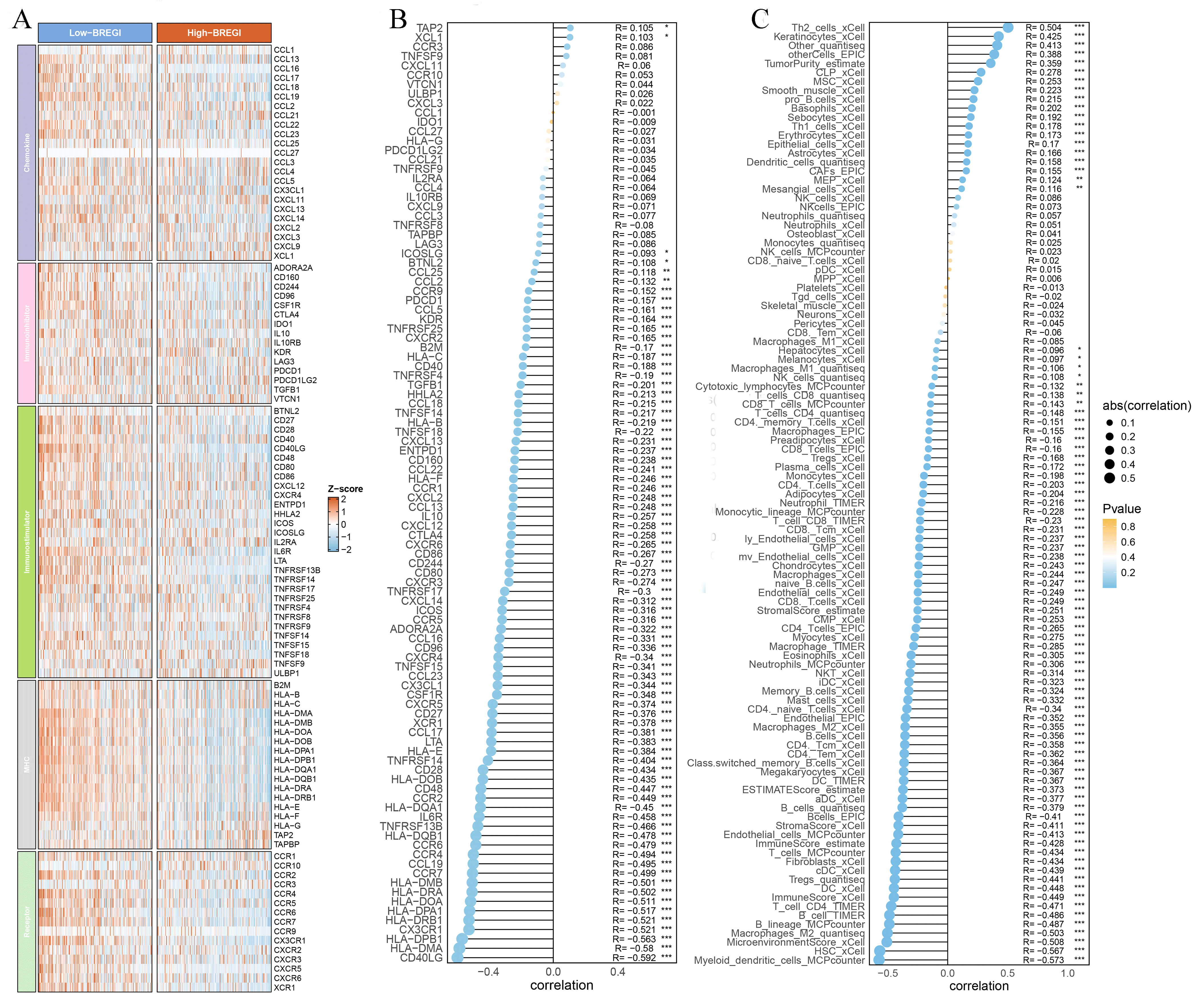


**Figure S4. Analyses of BREGI and immune-related factors.**

1. Expression of immune-related molecules in BREGI subgroups. (B) Correlation between BREGI and immune-related molecule expression. (C) Correlation between BREGI and immune cell populations.





**Figure S5. Genomic landscape differences between BREGI-high and BREGI-low groups in the TCGA dataset.** (A, B) Comparison of overall mutation frequencies between BREGI-high (A) and BREGI-low (B) groups. (C) Top 30 genes with the most pronounced differences in mutation frequency. (D) Co-mutation analysis of the top 30 genes. (E) Correlation analysis between BREGI expression and key genomic features. (F, G) Comparison of CNV events between BREGI-high (F) and BREGI-low (G) groups. (H, I) ChromPlot analysis showing that the G-scores of BREGI-high (H) and BREGI-low (I) groups.


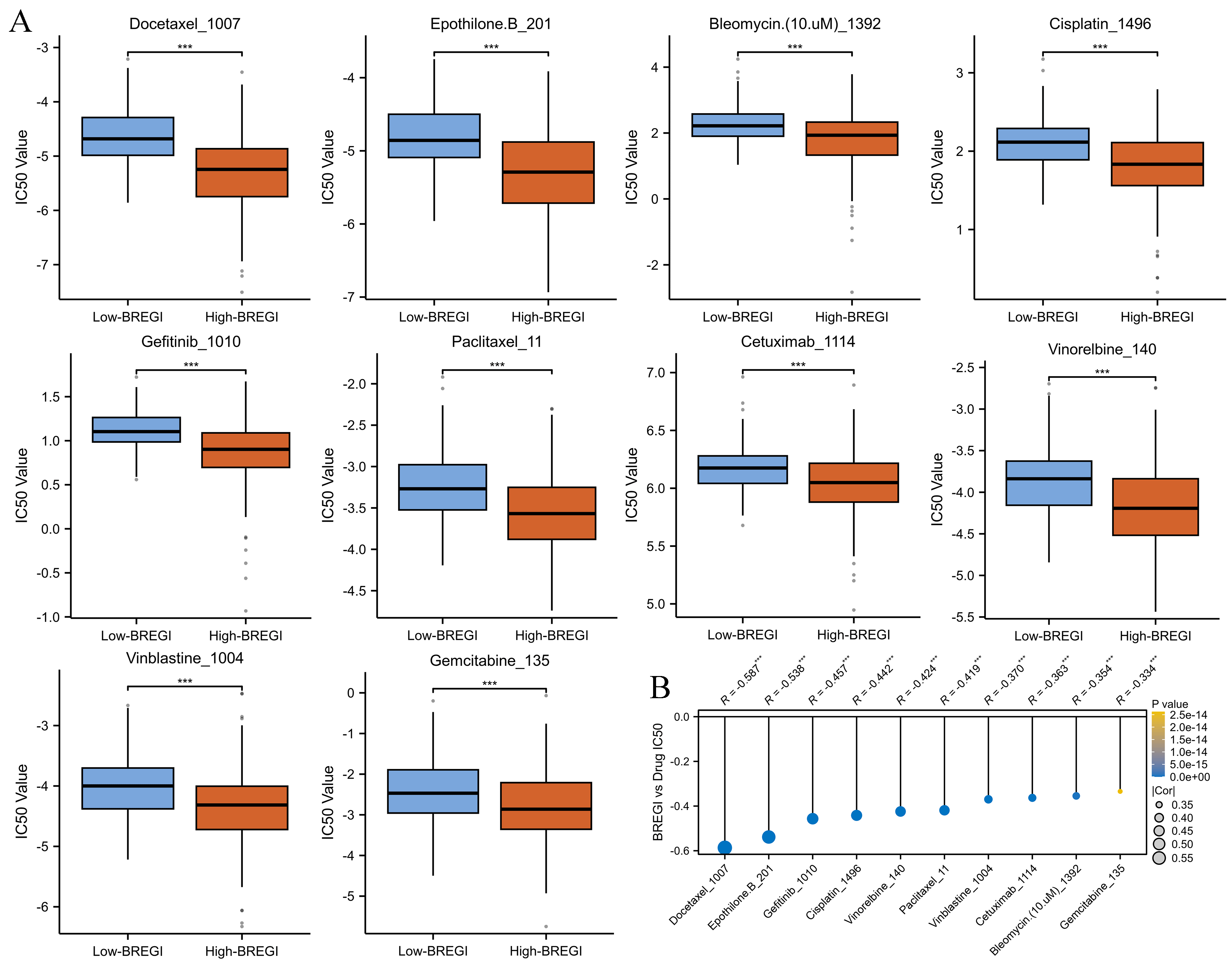


**Figure S6. Drug sensitivity analysis in BREGI subgroups.**

(A) Comparison of IC50 values for different drugs between high- and low-BREGI groups. (B) Correlation analysis between BREGI expression and drug IC50 values.
